# Has integrated medical insurance scheme enhanced happiness of returning migrant workers? Evidence from China

**DOI:** 10.3389/fpubh.2024.1467256

**Published:** 2024-11-20

**Authors:** Yupeng Shen, Yiming Meng, Yiyue Shan, Shichen Zhang, Daorui Tang, Zhengxiu Sun

**Affiliations:** ^1^School of Insurance, Shandong University of Finance and Economics, Jinan, China; ^2^School of Economics and Management, Southeast University, Nanjing, China; ^3^Department of Sociology and Anthropology, National University of Singapore, Singapore, Singapore

**Keywords:** integrated medical insurance scheme, returning migrant workers, happiness, time-varying DID, health disparities

## Abstract

Using four waves of panel data from the China Labor-force Dynamic Survey (CLDS) spanning from 2012 to 2018, regarding gradual reforms of the Integrated Medical Insurance Scheme (IMIS) in China as a quasi-experiment, we establish a time-varying difference-in-differences (DID) model to systematically examine the impact of IMIS on the happiness of returning migrant workers. Further, a structural equation model is employed to explore the potential mechanisms and analyze the heterogeneity of the effect. The results indicate that IMIS significantly enhances the happiness of returning migrant workers, which remains robust after a series of robustness checks. In addition, this effect operates through two potential mechanisms, economic effects and integration effects, which account for 25.59 and 29.31% of the total effect, respectively. Compared to the older generation, low-income, low-health human capital, and high-reimbursement level regions, the enhancement of happiness due to IMIS is more pronounced among the new generation, high-income, high-health human capital, and low-reimbursement level regions of returning migrant worker, which might exhibit a “pro-wealth” characteristic. The findings provide empirical support and policy implications for continuously improving the health insurance system, promoting the equalization of basic public services between urban and rural areas, and consistently enhancing the happiness of returning migrant workers.

## 1 Introduction

In recent years, with the transfer of industries across regions, there has been a large-scale trend of returning migrant workers (1, 2). According to statistics from the China Household Income Project (CHIP)^1^ in 2013, more than half of the migrant worker families living in cities plan to return to their hometowns within the next 5 years (1, 3). Compared to migrant workers who remain in urban areas, returning migrant workers possess higher levels of economic capital, human capital, and skills, and are more likely to engage in entrepreneurial activities (4). This can undoubtedly provide new momentum for rural economic development and contribute to achieving China’s goal of common prosperity.

**Figure 1:**
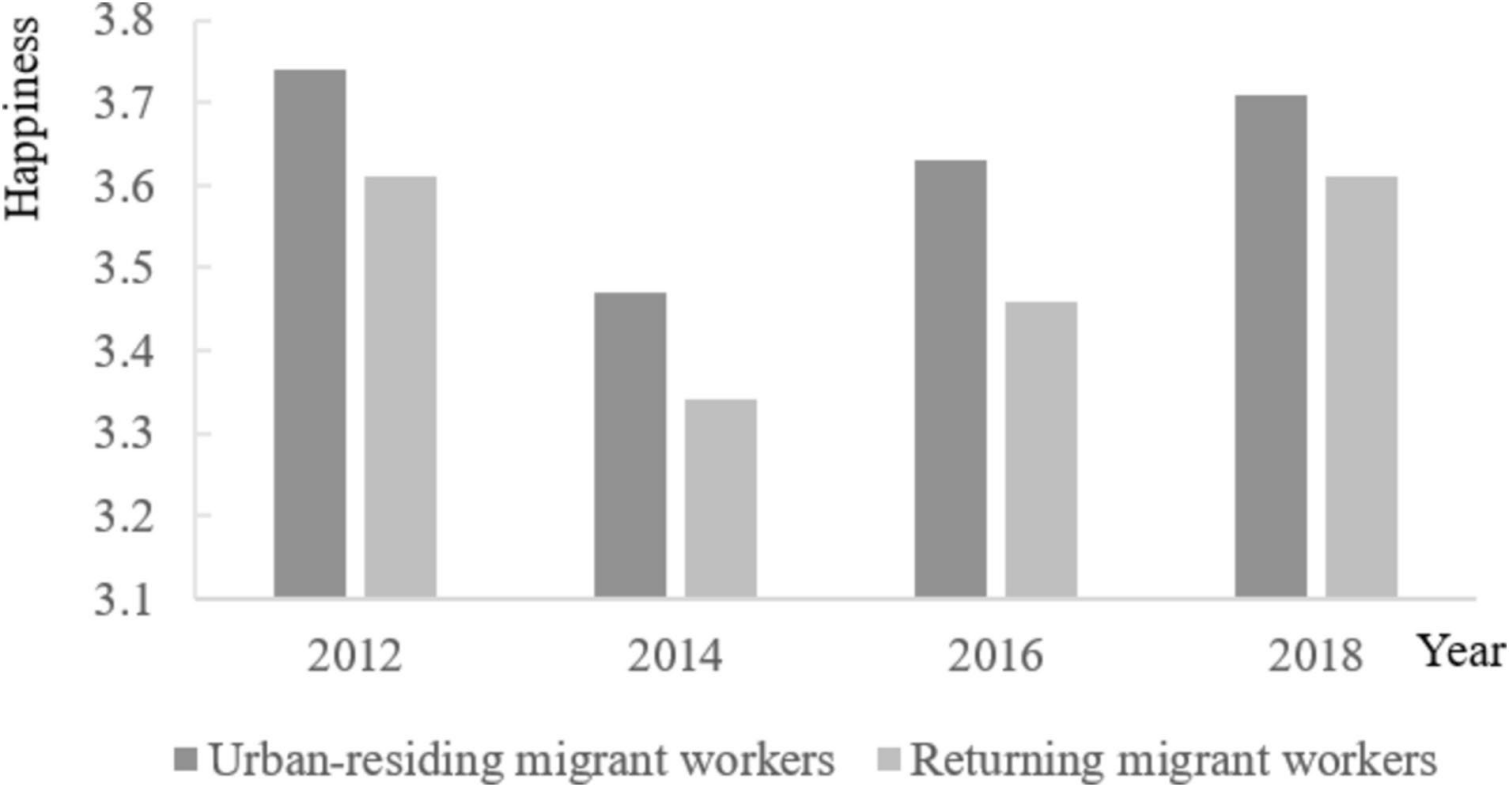
Happiness of urban-residing migrant workers and returning migrant workers.

However, as shown in Figure 1, returning migrant workers report lower levels of happiness compared to those who stay in urban areas, which is not conducive to improving employment probability and labor productivity (5). The primary reason is the perceived inequality and lack of fairness due to the urban–rural gap experienced during their time working outside their hometowns (6–10). In this context, as an important institutional reform to narrow the urban–rural gap, IMIS aims to break the fragmented social medical insurance system due to registered residence restrictions, ensure the equality of urban and rural residents in insurance opportunities, and improve the fairness of medical service utilization and health of urban and rural residents (11). So, we naturally raise the question of whether IMIS with the goal of promoting urban–rural equality will improve the relatively low happiness of returning migrant workers due to the urban–rural gap?

Theoretically, the rationale behind the impact of IMIS on happiness lies in two aspects: On the one hand, IMIS increases insurance subsidies for rural areas, improves the medical insurance benefits for returning migrant workers, thereby reducing their medical burden, relaxing financial constraints, and increasing their consumption of other goods. This economic effect can enhance the happiness of returning migrant workers. On the other hand, the strong love for hometown among migrant workers can enhance their sense of happiness when living in their hometown, but the urban–rural gap that returning migrant workers realize to some extent weakens this feeling. IMIS can narrow the urban–rural gap felt due to migrant work experience by breaking the institutional barriers of urban and rural registered residence restrictions and realizing the equalization of urban and rural medical service utilization, meet their recognition of their hometown, and enhance their willingness to live. This integration effect can improve the happiness of returning migrant workers. However, to the best of our knowledge, there is a lack of further discussion on the mechanisms by which IMIS affects subjective happiness. In addition, there are other shortcomings in existing research.

Firstly, in discussing subjective happiness, such as the happiness of rural residents, existing studies predominantly focuses on the general rural population (12–14), but overlooking the unique characteristics of the rapidly growing group of returning migrant workers within the context of new economic dynamics. This group exhibits distinct features and challenges in terms of employment, living conditions, and psychological dimensions, and the heterogeneity of their subjective experiences may serve as an intrinsic motivation influencing their employment decisions (14). Therefore, a deeper exploration of how IMIS impacts the subjective happiness of this group is crucial for understanding their employment behaviors and overall welfare.

Secondly, existing studies on the measurement and evaluation of subjective happiness, often relies on simplistic ordinal or dummy variables. This approach fails to fully uncover the underlying mechanisms through which health insurance policies affect individuals’ subjective experiences (15, 16). Such limitations not only restrict the depth and scope of the research but also raise concerns about the robustness and generalizability of the findings. Therefore, employing more comprehensive and scientifically rigorous measurement standards and evaluation methods to more accurately capture the impact of health insurance policies on the subjective happiness of returning migrant workers is a pressing issue for current research.

Since the non-uniform implementation timing of IMIS across different provinces in China, which exhibits a staggered pattern, this gives us an opportunity to explore the above issues under the time-varying difference-in-differences (DID) framework. This model is suitable for situations where the same policy is implemented progressively among affected groups. It not only controls for the impact of time-varying omitted variables but also assesses the policy’s effects by comparing differences before and after implementation, thus helping to determine the net effects of the policy. Therefore, using four waves of panel data from the China Labor-force Dynamic Survey (CLDS) spanning 2012, 2014, 2016, and 2018, regarding the progressive reform of China’s IMIS as a quasi-experiment, we establish a time-varying difference-in-differences (DID) model to examine the effects of this integration on the happiness of returning migrant workers. Furthermore, we develop a generalized mediation model within a structural equation framework to investigate the mediating mechanisms involved and test the heterogeneity of its effects.

We find that the IMIS significantly enhances the happiness of returning migrant workers, with an average increase of 7.42 percentage points. This effect remains robust after controlling for potential estimation biases using the conditional mixed process (CMP) method. Regarding potential mechanisms, we discover the roles of both economic and integration effects, which account for 25.59 and 29.31% of the total effect, respectively. In terms of heterogeneity analysis, compared to the older generation born before 1980, low-income individuals, those with low health human capital, and those in regions with high reimbursement levels, IMIS has a more significant impact on enhancing the happiness of the new generation born after 1980, high-income individuals, those with high health human capital, and those in regions with low reimbursement levels.

We may have the following marginal contributions: Firstly, we supplement the research on the policy effects of the IMIS. Previous studies have demonstrated the objective impacts of IMIS on issues such as the incidence of excessive labor among migrant workers (17), health shocks among residents (18), and the utilization of medical services (19, 20). Our focus is on the subjective happiness of returning migrant workers, thus expanding the understanding of the effects of the IMIS policy.

Secondly, we enrich the factors influencing happiness from the perspective of social security systems. Existing research has explored the positive effects of social policies like pension insurance (21, 22) and fertility policies (23) on happiness. However, to our knowledge, studies examining this issue from the perspective of improvements in the medical security system are still scarce. China’s IMIS inherently possesses attributes that mitigate inequality and promote income distribution (17, 24), which are crucial factors in enhancing happiness (25, 26). Our research confirms this point.

Thirdly, we enhance the understanding of the mechanisms through which social security policies affect individual happiness. We comprehensively analyze the dual roles of economic effects and social integration effects. Our findings show that IMIS not only improves the economic welfare of beneficiaries but also enhances their sense of social integration, thereby further promoting happiness. Additionally, the results reveal the “pro-wealth” characteristic of the policy, providing important policy implications for the precise design of future social security systems in China and the promotion of equalization of basic public services.

The subsequent sections are arranged as follows: Section 2 provides the policy background and a review of previous research. Section 3 details the empirical design. Section 4 presents the empirical results. Section 5 offers further analysis, and Section 6 concludes with a discussion.

## 2 Institutional background and previous studies

### 2.1 Medical insurance scheme

Before IMIS, Chinese residents participated in medical insurance according to their region of residence. Urban employees were covered under the Urban Employee Basic Medical Insurance (UEBMI), while urban residents participated in the Urban Resident Basic Medical Insurance (URBMI) and rural residents in the New Cooperative Medical Scheme (NCRMS). Although URBMI and NCRMS had similar payment standards and funding patterns, significant differences existed in the benefits received by participants based on their place of residence. Generally, URBMI offered higher reimbursement rates, a broader range of covered medical services, and higher caps for medical expenses compared to NCRMS (27, 28).

To achieve equitable medical insurance coverage for urban and rural residents and to reduce the substantial differences in medical service utilization between these groups, the State Council issued the “Opinions on Integrating the Basic Medical Insurance System for Urban and Rural Residents” in 2016. Following this directive, local governments began the process of integrating URBMI and NCRMS, with most regions implementing the integrated system by 2017. In fact, prior to 2016, some regions, such as Tianjin, Qinghai, Shandong, Chongqing, Guangdong, Ningxia, and Zhejiang, had already started exploring and successfully establishing unified medical insurance systems for urban and rural residents. By 2020, all 34 provinces in China had essentially completed the institutional integration of basic medical insurance for urban and rural residents.

The IMIS achieved “six unifications”: unified coverage, unified funding policies, unified benefit packages, unified medical insurance directories, unified designated medical institution management, and unified fund management. As of the end of 2023, the number of people enrolled in the Urban and Rural Resident Basic Medical Insurance (URRBMI) reached 962.93 million. The advancement of IMIS is regarded as a significant measure by the Chinese government to narrow the urban–rural gap and enhance universal health coverage.

### 2.2 Previous studies

#### 2.2.1 Research on the influencing factors of happiness

Factors influencing happiness can be summarized into three categories: demographic characteristics, individual behavioral decisions, and social policies and environments. In terms of demographic characteristics, Blanchflower (16) found a U-shaped relationship between age and happiness using data from 145 countries. The likely reason is that individuals experience higher stress and weaker resilience during midlife, resulting in the lowest subjective happiness (29). Concerning health, most studies find that satisfaction with health, physical health, and mental health all impact happiness positively (30). Specifically, Munasinghe et al. (31), in the context of the COVID-19 pandemic, found that the frequency of outdoor activities and outdoor surveys could mitigate the impact of health risks on happiness to some extent. Additionally, extensive literature indicates that individuals who are married, have a higher level of education, or possess attractive facial features are more likely to experience higher levels of happiness (5, 32–35). However, regarding the relationship between gender and happiness, current research has not reached a consensus. Carlson and Hans (36), from the perspective of household labor division, found that women bearing a disproportionate share of domestic responsibilities tend to have lower happiness. Conversely, Becchetti and Conzo (15) argue that women, despite having lower resilience compared to men, exhibit stronger subjective happiness.

For individual behavioral decisions, income is one of the key determinants of happiness. The relationship between income and happiness predominantly builds on the “Easterlin Paradox” (37, 38), with further exploration by some scholars from the perspectives of relative and absolute income. Absolute income, which fulfills physiological and safety needs, demonstrates a positive link with happiness (39). Conversely, due to a comparative mentality, larger disparities in relative income tend to reduce overall happiness (40, 41). However, there are also some studies that show no correlation between income and happiness (42). Employment also plays a role, where the risk of unemployment negatively correlates with happiness (43), and high-quality employment boosts happiness (44). Additionally, fertility enhances happiness, having children generally increases happiness (45), and a higher number of children is associated with greater happiness (46). Meanwhile, the gender of children can somewhat influence parental happiness during their upbringing (47).

Regarding social policy and environment, government investment in public services such as education and healthcare significantly influences residents’ happiness (48). As a pivotal social policy, the social security system inherently possesses the capacity to mitigate uncertain risks, directly enhancing residents’ happiness (29). Hou (49) found that individuals with pension and health insurance exhibit stronger subjective happiness compared to those without such insurance. Research by Bairoliya et al. (25) indicates that comprehensive government-provided healthcare and older adult care services reduce individual precautionary savings, increase consumption, and enhance happiness.

Meanwhile, the social security system also serves a role in income distribution, mitigating the changes in happiness caused by income disparities. Studies by Ma and Xi (26) demonstrate that social security can alleviate the unfairness caused by income disparities, improve individual consumption and health levels, and consequently increase feelings of happiness and fulfillment. Additionally, social networks contribute to individual happiness through economic and emotional exchanges (12), and the degree of social integration also plays a crucial role in influencing individual happiness (50).

#### 2.2.2 Research on the policy effects of IMIS

In the context of China’s dual urban–rural system, a dual-track medical insurance model has emerged, operating both the NRCMS and Urban Resident Basic Medical Insurance (URBMI), leading to issues such as unfair treatment and mobility lock-in (51, 52). In response, China has been experimenting with the integration of urban and rural medical insurance, known as the IMIS, which has garnered extensive scholarly attention for its real-world effects.

In terms of healthcare services, significant research demonstrates that, adhering to the principles of “higher benefits rather than lower, broader scope rather than narrower,” the implementation of IMIS has notably increased medical service utilization among rural residents (53), and substantially decreased the occurrence of untreated illnesses (54), effectively mitigating health and medical service inequalities across different economic strata (55).

In labor decision-making, Li et al. (17) explored the effects of the IMIS from the perspective of interest equalization, revealing that IMIS bolstered migrant workers’ capabilities to withstand health and economic risks, thereby easing the burden of excessive labor. Additionally, Hong and Ning (56) examined the portability of benefits following the implementation of IMIS, finding that it incentivized rural residents to pursue job opportunities within urban areas. Notably, Zheng et al. (18) discovered that IMIS reduced individuals’ out-of-pocket medical expenses and increased labor supply, thus facilitating targeted poverty alleviation, especially among those affected by health risks.

Overall, the improvements in health status, equity, and accessibility of healthcare services due to IMIS have significantly raised the happiness of rural residents (57). The results of the above studies all support the idea that the integration of medical insurance systems not only improves access to healthcare but also has a positive impact on labor decisions, economic welfare, and overall quality of life for rural and migrant populations.

#### 2.2.3 Research on the relationship between IMIS and the happiness of returning migrant workers

Returning migrant workers often experience lower levels of happiness due to perceived inequalities stemming from the urban–rural divide felt during their time working away from home (58). The Integrated Medical Insurance Scheme (IMIS) serves as a crucial mechanism for equalizing public services, aiming to dismantle the segmentation of the social health insurance system caused by household registration restrictions and to promote equality between urban and rural areas. This, to an extent, addresses the gaps in social security for migrant workers (17), and also facilitates the enhancement of happiness among returning migrant workers. Specifically, IMIS enhances the happiness of returning migrant workers through the following two aspects:

Firstly, the essence of medical security lies in pooling public finance and individual premiums to form a risk fund, which is ultimately distributed to those who are ill and to groups who cannot access medical services. This process facilitates the transfer of funds from healthy to unhealthy individuals and from high-income to low-income groups, achieving the purpose of income redistribution (57). However, under the dual urban–rural system, the New Rural Cooperative Medical Scheme (NRCMS) has historically been characterized by low funding levels and low protection levels, making health shocks a greater financial threat, with the “happiness effect” of medical security not being pronounced.

Under the principle of “ensuring higher rather than lower benefits, and broader rather than narrower coverage” (59), the IMIS policy has increased subsidies for rural healthcare and raised the level of coordination, especially ensuring equal treatment with urban residents. This has greatly enhanced the scope and level of protection, thereby reducing the medical burden on returning migrant workers compared to the NRCMS period, easing financial constraints, continuously improving economic welfare, and subsequently increasing consumption of other goods that improve life quality, ultimately enhancing overall utility.

Secondly, migrant workers possess a strong attachment to their rural roots and cultural identity (60). Growing up in rural areas instills in them a deep-seated customary inertia, with emotional bonds rooted in kinship, clan, and land, forming a distinctive local identity and fondness for their native soil. In other words, compared to relocating to cities, migrant workers experience higher satisfaction living in their native rural environments (61). However, the perceived urban–rural disparity felt by returning migrant workers due to their experiences working in cities can lead to discomfort with rural life, reducing their happiness, and the direct consequence of this perceived disparity often results in their settlement in urban areas (62).

The goal of the IMIS is to equalize the utilization of urban and rural medical services by breaking down the institutional barriers imposed by the urban–rural household registration system, a critical component of public service equalization. Therefore, for returning migrant workers, IMIS narrows the perceived urban–rural gap experienced due to their work outside, facilitates their reintegration into their native rural areas, and thus enhances their happiness through a strong sense of local identity and attachment to their native land.

Based on the foregoing discussion, we propose the following research hypotheses:

*H1*: The IMIS enhances the happiness of returning migrant workers.

*H2*: The IMIS improves the happiness of returning migrant workers through economic effects and integration effects.

## 3 Empirical strategy

### 3.1 Data

The data used in this paper are drawn from 4 waves of the CLDS conducted in 2012, 2014, 2016, and 2018. The CLDS, conducted by the Social Science Survey Center at Sun Yat-sen University, is China’s first interdisciplinary, nationwide longitudinal survey focused on labor. It examines the current status and changes in China’s labor force, covering a wide range of research topics including education, employment, migration, health, social participation, economic activities, and grassroots organizations. The CLDS establishes a comprehensive database centered on labor by conducting biennial tracking surveys in urban and rural communities across China. This database includes longitudinal and cross-sectional data at the individual, household, and community levels.

The CLDS sample is representative and diverse, with each survey round covering at least 28 provinces and municipalities. The sample size includes over 400 communities, more than 14,000 households, and over 20,000 labor force individuals. It employs a multi-stage, multi-level probability sampling method proportional to labor force size and is among the first in China to use a rotating sample tracking approach. This method effectively adapts to China’s rapidly changing environment while maintaining the characteristics of cross-sectional surveys, providing high-quality foundational data for empirically oriented theoretical and policy research. We focus on the happiness of returning migrant workers, considering the IMIS as an exogenous policy shock.

The reasons for selecting this dataset are as follows: Firstly, the data have precise city codes that accurately identify the areas with IMIS, providing the necessary conditions to establish treatment and control groups required for the DID method used in this study; secondly, the survey targets the working population aged 15 to 64, detailing their employment conditions, which allows for accurate identification of the returning migrant worker within the labor force; thirdly, the dataset includes individual, family, and community data, covering a wide range of information such as employment details, educational background, mobility intentions, family economics, and community relationships, which comprehensively meets the data requirements of this study. Additionally, the IMIS data employed in this paper are compiled from various regional policy documents organized by the authors.

In line with the research objectives, the data were processed as follows: First, only samples of returning rural migrant workers were retained, specifically those who had previously worked outside their home county and had returned to their registered residence by the time of the survey with no intention of migrating again. Second, samples of individuals aged 16 to 64, representing the working-age labor force, were retained. Third, only those who participated in either the NRCMS or URRBMI during the survey period were included. Fourth, observations with missing key variables were excluded. Ultimately, 4,616 valid observations were retained.

### 3.2 Empirical model

Given the gradual implementation of the IMIS in urban and rural areas across China, following Beck et al. (63), we adopt the time-varying DID approach to evaluate the impact of the IMIS on the happiness of returning migrant workers. Specifically, regions where IMIS has been implemented are designated as the treatment group, while the others serve as the control group. The year of IMIS implementation in the treatment regions and all subsequent years are defined as the treatment period, with all other years considered the control period. This setup allows for an accurate assessment of the policy’s effects by examining the differences in happiness between the treatment and control groups before and after the policy shock. Moreover, as the dependent variable, *Happiness*, is an ordinal variable ranging from 1 to 5, we employ an Order-Probit model for empirical analysis, which is as follows:


(1) $$\begin{array}{l} \mathrm{Pr}\left(\mathrm{Happines}s_{ikt}\right)=\beta_{0}+\beta_{1}IMIS_{ikt}+\underset{m}{\sum }\alpha_{m}X_{ikt}^{m}\\ \;+\gamma_{t}+\lambda_{k}+\gamma_{t}\times \lambda_{k}+\mu_{ikt} \end{array}$$

where the outcome variable is $\mathrm{Happines}s_{ikt}$ , which indicates the ordered level of individual happiness of individual i in the city k in year t . It ranges from 1 to 5, with higher values indicating higher levels of happiness. The independent variable is $IMIS_{ikt}$ , which is a dummy variable that equals 1 if the individual i ‘s city k implements the IMIS in year t , and 0 if otherwise. $X_{ikt}^{m}$ is a series of individual-level, household-level and village-level control variables. $\beta_{0}$ is the constant term. $\beta_{1}$ is the coefficient of the core explanatory variable. When $\beta_{1}$ >0, it indicates that the IMIS increases the happiness of rural migrant workers, otherwise it decreases happiness. $\alpha_{m}$ denotes the estimated coefficients of control variables. $\gamma_{t}$ denotes year fixed effects. $\lambda_{k}$ denotes city fixed effects. $\gamma_{t}\times \lambda_{k}$ denotes city-year fixed effects. $\mu_{ikt}$ is the error term.

### 3.3 Variable selection and measurement

Dependent variable: The dependent variable (*Happiness*) in the baseline regression is an ordinal variable representing individual happiness, ranging from 1 to 5. Specifically, 1 indicates “very unhappy,” 2 indicates “unhappy,” 3 indicates “neutral,” 4 indicates “happy,” and 5 indicates “very happy”.

Independent variable: The key independent variable (*IMIS*) is a binary dummy variable indicating the IMIS. Based on regional policy documents, the value is set to 1 if the region implemented the IMIS policy during the corresponding year, and 0 otherwise.

Control variables: Referring to Cheng and Hua (29), and Luo and Liu (57), which explore the determinants of happiness among rural residents, the following control variables are included:

1. Individual-level characteristics:

These variables include *Age, Age^2^, Gender, Marital status, Education level, Party, Income level, Working hours, Job type, Health status,* and *Pension insurance. Age* is measured as the difference between the survey year and birth year, and *Age^2^* is added to account for the nonlinear effect of age on happiness. *Gender* is a dummy variable, where male = 1 and female = 0. *Marital status* is a dummy variable, where married, remarried, or cohabiting = 1, and unmarried, divorced, or widowed = 0. *Education level* is an ordinal variable ranging from 0 to 5, corresponding to never attended school, primary school, middle school, high school (including technical schools), undergraduate (including vocational schools), and postgraduate (master’s or doctoral degrees). *Party* is a dummy variable, with Communist Party members coded as 1, and others as 0. *Income level* is the natural logarithm of individual annual income. *Working hours* are the natural logarithm of the individual’s total annual working hours. *Job type* is a dummy variable, where employment in public sectors such as government agencies or state-owned enterprises = 1, and others = 0. *Health status* is an ordinal variable ranging from 1 to 5, representing “very healthy,” “healthy,” “neutral,” “unhealthy,” and “very unhealthy.” *Pension insurance* is a dummy variable, with a value of 1 if the individual has either basic pension insurance or commercial pension insurance, and 0 otherwise.

1. Household-level characteristics:

These include the *Number of dependents*, *Home ownership*, and *Family harmony*. *Number of dependents* refers to the total number of individuals in the household under the age of 16 or over 65. *Home ownership* is a dummy variable, with 1 indicating ownership of a house, and 0 otherwise. *Family harmony* is measured as an ordinal variable ranging from 1 to 5, with 1 indicating “very disharmonious,” 2 indicating “disharmonious,” 3 indicating “neutral,” 4 indicating “harmonious,” and 5 indicating “very harmonious.”

1. Village-level characteristics:

These include *Village harmony* and *Per capita fiscal revenue of village*. *Village harmony* is an ordinal variable ranging from 1 to 5, where 1 represents “very disharmonious,” 2 represents “disharmonious,” 3 represents “neutral,” 4 represents “harmonious,” and 5 represents “very harmonious.” *Per capita fiscal revenue of village* is calculated as the total fiscal income of the village divided by the number of residents, and the natural logarithm of this value is used in the analysis.

### 3.4 Descriptive statistics

Table 1 reports the descriptive statistics of the variables, including the means and standard deviations for the full sample, as well as subsamples for the control and treatment groups. Additionally, the differences in means between the subsamples were calculated. The results show that, over the entire survey period, 44.37% of the sample resided in regions where the IMIS had been implemented. Moreover, there is a significant difference in happiness between returning rural migrant workers in the treatment and control groups.

**Table 1:** Descriptive statistics.

Variables	(1) Full sample	(2) Control group	(3) Treatment group	(4) (3)–(2)
Happiness	3.5043 (1.01)	3.3586 (1.06)	3.6870 (0.93)	0.3284***
IMIS	0.4437 (0.50)			
Age	44.9887 (12.29)	43.8555 (12.33)	46.4097 (12.08)	2.5541***
Age^2^	21.7487 (11.14)	20.7522 (11.03)	22.9982 (11.15)	2.2460***
Gender	0.6163 (0.49)	0.6059 (0.49)	0.6294 (0.48)	0.0235
Marital status	0.9084 (0.29)	0.9050 (0.29)	0.9126 (0.28)	0.0076
Education level	1.5084 (0.89)	1.3910 (0.89)	1.6558 (0.87)	0.2648***
Party	0.0479 (0.21)	0.0514 (0.22)	0.0435 (0.20)	−0.0079
Income level	6.6289 (4.36)	6.1496 (4.38)	7.2299 (4.26)	1.0802***
Working hours	6.1440 (2.62)	6.1989 (2.54)	6.0751 (2.71)	−0.1238
Job type	0.0609 (0.24)	0.0600 (0.24)	0.0620 (0.24)	0.0020
Health status	2.5104 (1.02)	2.5000 (1.04)	2.5234 (0.98)	0.0234
Pension insurance	0.5604 (0.50)	0.5833 (0.49)	0.5317 (0.50)	−0.0516***
Number of dependents	1.7444 (1.40)	1.6935 (1.32)	1.8081 (1.50)	0.1146***
Home ownership	0.9289 (0.26)	0.9338 (0.25)	0.9229 (0.27)	−0.0109
Family harmony	3.3967 (0.65)	3.5471 (0.73)	3.2080 (0.46)	−0.3391***
Village harmony	3.1597 (0.46)	3.2531 (0.58)	3.0425 (0.20)	−0.2106***
*Per capita* fiscal revenue of village	0.1532 (0.77)	0.2063 (0.89)	0.0865 (0.59)	−0.1198***
Observation	4,616	2,568	2048	4,616

Column (4) tests the significance of sample differences using t-statistics; ***, **, and * indicate statistical significance at the 1, 5, and 10% levels, respectively. Standard deviations are reported in parentheses.

## 4 Empirical results

### 4.1 Benchmark results

Table 2 presents the regression results of the impact of the IMIS on the happiness of returning migrant workers. Columns (1) to (3) employ the Ordered Probit model, varying in the inclusion of control variables and fixed effects: column (1) presents results without any controls or fixed effects, column (2) includes fixed effects, and column (3) incorporates both fixed effects and all control variables. The results indicate that the coefficient for IMIS is significantly positive at the 1% statistical level across all specifications. Given that coefficients estimated by the Ordered Probit model do not have a direct economic interpretation, the study further calculates the marginal effects of this variable, which are 0.0858, 0.0753, and 0.0742, respectively. These findings suggest that the IMIS increases the happiness of returning migrant workers by approximately 7.42 to 8.58 percentage points.

**Table 2:** Benchmark results.

	(1)	(2)	(3)	(4)	(5)
	Order Probit	Order Probit	Order Probit	OLS	Probit
IMIS	0.3397***	0.3068***	0.3179***	0.2734***	0.6046***
	(10.79)	(3.50)	(3.58)	(3.49)	(3.71)
Age			−0.0202***	−0.0179***	−0.0281***
			(−12.49)	(−12.67)	(−10.31)
Age^2^			0.0246*	0.0206*	0.0380**
			(1.78)	(1.89)	(2.29)
Gender			−0.0416	−0.0393	−0.0656
			(−1.14)	(−1.22)	(−1.11)
Marital Status			0.2636***	0.2347***	0.3212***
			(4.65)	(4.65)	(3.73)
Education Level			0.0797***	0.0711***	0.1012***
			(3.69)	(3.71)	(2.84)
Party			0.3073***	0.2571***	0.3634**
			(3.94)	(3.79)	(2.28)
Income Level			0.0074	0.0066	0.0107
			(1.43)	(1.45)	(1.30)
Working Hours			−0.0077	−0.0061	−0.0014
			(−1.06)	(−0.95)	(−0.12)
Job Type			−0.0269	−0.0236	−0.0888
			(−0.37)	(−0.37)	(−0.75)
Health Status			−0.2572***	−0.2241***	−0.3256***
			(−14.91)	(−14.91)	(−11.47)
Pension Insurance			0.0537	0.0498*	0.1202**
			(1.57)	(1.65)	(2.15)
Number of Dependents			−0.0131	−0.0121	−0.0428**
			(−1.12)	(−1.17)	(−2.24)
Home Ownership			−0.0834	−0.0765	−0.0527
			(−1.33)	(−1.37)	(−0.53)
Family Harmony			0.0490	0.0446	0.1303**
			(1.56)	(1.60)	(2.54)
Village Harmony			−0.0329	−0.0263	−0.0191
			(−0.52)	(−0.48)	(−0.20)
*Per Capita* Fiscal Revenue of Village			0.0001*	0.0001*	0.0007
			(1.71)	(1.69)	(1.31)
Year FE		Yes	Yes	Yes	Yes
City FE		Yes	Yes	Yes	Yes
Year × City FE		Yes	Yes	Yes	Yes
Constant				3.6113***	−0.2655
				(4.43)	(−0.37)
*R*^2^/Pseudo *R*^2^	0.0127	0.0290	0.0617	0.1600	0.1545
Observation	4,616	4,616	4,616	4,616	4,214

The significance levels of 1, 5, and 10% are denoted by ***, **, and *, respectively. Standard errors are clustered at the year-city level. The t-values are in parentheses. Same below.

The OLS regression results are shown in column (4). The estimated coefficient of the IMIS remains positive and statistically significant at the 1% level. Furthermore, drawing on prior literature on the design of happiness indicators (64), we redefine the happiness variable as a dummy variable: respondents who answer “unhappy” or “very unhappy” equal 0, and 1 if otherwise. Using the Probit model, we present the result in Column (5), which remains robust. These findings confirm Hypothesis 1.

### 4.2 Endogeneity analysis

This study employs the implementation of IMIS at the hometowns of returning migrant workers as the core explanatory variable. The sample selection is confined to returning migrant workers whose *hukou* matches their current residence and who consistently participated in the health insurance during the survey period. This approach substantially mitigates issues of reverse causality and sample selection bias. However, while changes in health insurance policies are relatively exogenous governmental decisions (65), they also result from negotiations among various levels of government (66), leading to some endogeneity due to differences in integration patterns and levels across regions. Additionally, returning migrant workers, having spent extended periods working away from home, may have limited understanding of local health insurance policies, which potentially underestimates the impact of the IMIS on their happiness. Moreover, as happiness is influenced by multiple factors, it is challenging for models to control for all potential influences. These uncontrolled factors could simultaneously affect the happiness of returning migrant workers and the outcomes of health insurance policies, leading to endogeneity issues due to omitted variables.

To address potential regression biases, we employ the instrumental variable (IV) method for analysis. The principle of IV method is to identify a variable that is strongly correlated with the primary explanatory variable in the model but is as uncorrelated as possible with the error term. By incorporating this variable into the model transformation, a consistent estimator of the relevant parameters is constructed alongside other variables, enhancing the precision of the estimation. Given that the IMIS variable is a dummy, traditional IV-Probit models, which are suited primarily for continuous endogenous variables, are not applicable. Referring to Roodman (67) and Li et al. (17), we employ the conditional mixed process (CMP) estimation for a two-stage regression. In the first stage, the CMP-probit method is used to examine the relationship between the IV and the endogenous variable. The second stage uses the CMP-oprobit method to assess the impact of IMIS on the happiness of returning migrant workers. Furthermore, the endogeneity of the IMIS policy is identified through the atanhrho_12 parameter, which serves as an endogeneity test.

Referring to Li et al. (17), we select “*per capita* public fiscal revenue” and “proportion of the population in municipal districts” of the cities where returning migrant workers reside as IVs. The former is defined as the city’s public fiscal revenue for the year divided by the total population of the city, and then taking the logarithm. The latter is defined as the number of people in the urban area divided by the total population of the city. Before reporting the regression results, it is crucial to analyze the relevance and exogeneity of these instruments. In terms of relevance, the capability of public finance is a crucial guarantee for the government to equalize basic public services (68), and *per capita* public fiscal revenue directly reflects local public financial capability. Higher public financial capacity increases the likelihood of implementing IMIS. The proportion of the population in municipal districts can, to some extent, reflect the relative size of the rural and urban registered populations, further revealing differences in the scale of participation in the NRCMS and Urban Resident Basic Medical Insurance (URBMI).

Given that China’s IMIS practices often involve extending the relatively lower benefits of the former to match those of the latter—with the shortfall primarily funded by fiscal means (69)—a higher population proportion in municipal districts suggests a larger scale of urban insurance participation, hence less fiscal pressure during integration, making IMIS implementation more feasible. Thus, the chosen IVs are relevant to the endogenous variable. Regarding exogeneity, neither of them is likely to directly affect the happiness of returning migrant workers or their personal and family characteristics. Similarly, the happiness of these workers is unlikely to directly influence regional fiscal capabilities or population size, fulfilling the requirements for the exogeneity of the IVs.

The results of the two-stage regression using CMP are presented in Table 3. In the first stage regression, the coefficients for both *per capita* public fiscal revenue and the proportion of the population in municipal districts are positive and significant at the 1% statistical level. This demonstrates that higher regional public financial capabilities and a larger relative scale of urban insurance participation facilitate the implementation of the IMIS, further validating the relevance of the instrumental variables. In the second stage regression, the atanhrho_12 value is nonzero and significant at the 5% statistical level, indicating some degree of endogeneity in IMIS. After correcting for endogeneity bias, the coefficient for the IMIS variable remains positive and significant at the 1% statistical level.^2^

**Table 3:** CMP estimation results.

Variables	Stage1	Stage2
	Convergence and integration	Happiness
IMIS		0.4326*** (2.82)
Revenue	0.1159*** (14.12)	
Percentage	0.1413*** (3.50)	
Atanhrho_12		0.4500** (2.27)
Control variables	Yes	Yes
Year FE	Yes	Yes
City FE	Yes	Yes
Year × City FE	Yes	Yes
Observation	4,327	4,327

### 4.3 Robustness checks

#### 4.3.1 Parallel trend test

**Figure 2:**
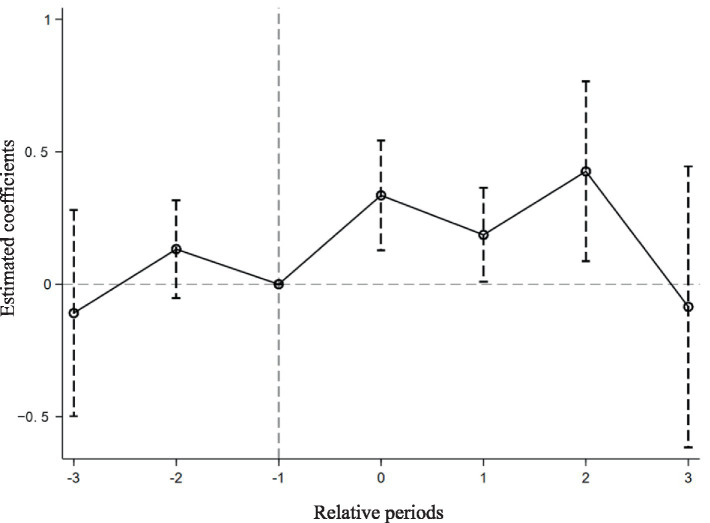
Parallel trend test.

The prerequisite for DID approach is the satisfaction of the parallel trends assumption, which posits that, prior to the implementation of the IMIS, both the treatment and control groups exhibit similar trends in happiness. Due to the asynchronous timing of IMIS implementation across cities, traditional DID methods for testing parallel trends cannot directly observe the changes in happiness between the groups. Thus, following Beck et al. (63), we use an event study methodology to test for parallel trends. This method provides the advantage of directly observing whether the treatment and control groups share similar trends prior to the implementation of IMIS and allows for the analysis of the dynamic effects of IMIS on the happiness of returning migrant workers. Figure 2 demonstrates the point estimates and confidence intervals for the average treatment effects relative to the period just before IMIS implementation.

It can be observed that before IMIS, policy effect estimates fluctuate around zero, and confidence intervals include zero, indicating no significant pre-existing differences in happiness between the groups, thereby confirming the parallel trends assumption. Subsequently, from the onset of IMIS implementation, the policy effect estimates diverge significantly from zero, and the confidence intervals no longer include zero, clearly demonstrating that IMIS significantly enhances the happiness of the treatment group of returning migrant workers with a noticeable policy persistence effect. It is noteworthy that in the third post-implementation period, the policy effect is not significant, likely due to limited data. Observations for the post-three periods are only available for the year 2012 when few areas had implemented IMIS, leading to greater variance and less precise estimates. Even excluding data limitations, the improvements in rural health insurance benefits following IMIS implementation may have met the medical needs of returning migrant workers over time, thus diminishing the observable effects of the policy.

#### 4.3.2 Placebo test

**Figure 3:**
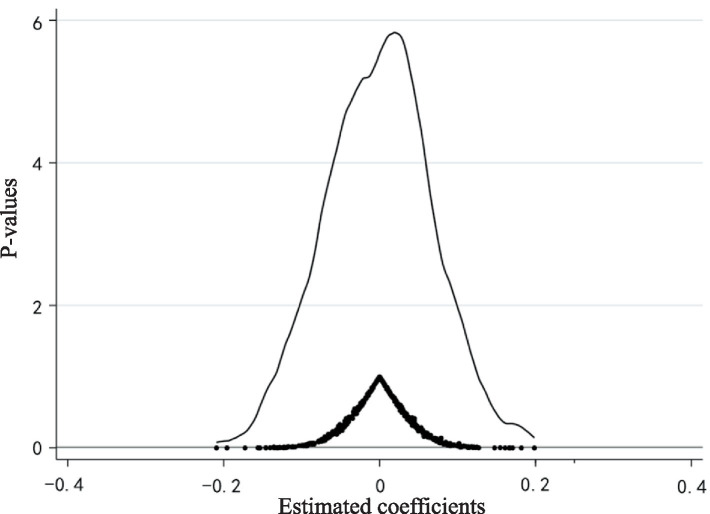
Placebo test.

Although this study includes time-by-region interaction fixed effects to control for factors that change over time within regions, data limitations still pose a risk that some city-year characteristics remain unobservable. Consequently, changes in the happiness of returning migrant workers might be attributed to other factors, suggesting potential spurious correlations in the baseline model. Following the method proposed by Yu et al. (70), we employ a placebo test using a random assignment of treatment groups. Specifically, for each year that the IMIS was actually implemented, an equivalent number of cities are randomly selected to form a fictitious treatment group. If significant improvements in the happiness of returning migrant workers are observed in these fictitious treatment areas, it would indicate that the observed improvements are not solely attributable to IMIS, rendering the regression results unreliable. According to this method, we conduct 500 random draws and regresses using Equation 1, producing a distribution of estimated coefficients for the IMIS variable and their corresponding *p*-values, as shown in Figure 3. The results indicate that the estimated coefficients from the random samples are predominantly clustered around zero and are lower than those from the baseline regression, with corresponding p-values generally higher than those of the baseline regression coefficients (0.0500). Therefore, it is plausible to largely discount the influence of unobservable factors on the regression results, affirming that IMIS genuinely enhances the happiness of returning migrant workers.

#### 4.3.3 Alternative specifications

While our measures of happiness are supported by numerous studies (64, 71), some studies use life satisfaction as an indicator of happiness (72, 73). Therefore, based on the question from the CLDS survey, “Overall, are you satisfied with your living conditions?,” we use life satisfaction as a proxy for happiness, as shown in Column (1) of Table 4. Additionally, we use economic satisfaction, derived from the question, “Overall, are you satisfied with your family’s economic situation?,” as another proxy for happiness, with results presented in Column (2). It is observed that regardless of the happiness measure used, the coefficient for IMIS variable is positive and significant at the 1% statistical level.

**Table 4:** Alternative specifications.

	(1)	(2)	(3)
Variables	Life satisfaction	Economic satisfaction	Happiness
IMIS	0.3897*** (4.22)	0.2914*** (3.20)	
URRBMI			0.4109** (2.12)
Control variables	Yes	Yes	Yes
Year FE	Yes	Yes	Yes
City FE	Yes	Yes	Yes
Year × City FE	Yes	Yes	Yes
*R*^2^/Pseudo *R*^2^	0.0709	0.0663	0.0822
Observation	3,742	3,742	3,742

Additionally, we refine the core explanatory variable based on self-reported health insurance types from respondents in the CLDS. Specifically, we create a dummy variable, *URRBMI*, which equals 1 if the respondent indicates having “Urban and Rural Residents Basic Medical Insurance” according to the questionnaire item asking “Which of the following medical insurances/protections do you have?” and equals 0 if the answer is “New Rural Cooperative Medical Scheme.” We then re-estimate Equation 1 with this revised variable. The results, as shown in Column (3) of Table 4, indicate that the coefficient for the IMIS variable is 0.4109, significant at the 5% statistical level. This demonstrates that, even after changing the method for setting the core explanatory variable, the IMIS continues to significantly enhance the happiness of returning migrant workers.

## 5 Further analysis

### 5.1 Potential mechanism

Existing research indicates that returning migrant workers experience lower happiness compared to rural workers who remain in their hometowns. The primary reasons are a dual decline in income satisfaction and a sense of social equity, with the latter mainly stemming from the perceived urban–rural disparity acquired through their experiences working away from home (74). Therefore, this section conducts an in-depth theoretical analysis to further explore the impact of economic and integration effects on the happiness of returning migrant workers, and quantitatively analyze the mechanisms and specific magnitudes of these effects.

Although previous researchers typically used mediation models to study this issue, there are endogeneity issues with mediation models, which are reflected in the following three aspects. Firstly, although random experiments may ensure exogeneity, it is very easy for estimation coefficients to be biased due to confounding factors that affect both mediator and outcome variables. Secondly, the measurement error of the mediator variable leads to a zero bias in the coefficient of influence of the mediator variable on the dependent variable, ultimately resulting in an overestimation of the coefficient of the core explanatory variable on the dependent variable. Thirdly, the causal direction in mutual causality can also lead to estimation errors in coefficients in different directions (75).

**Figure 4:**
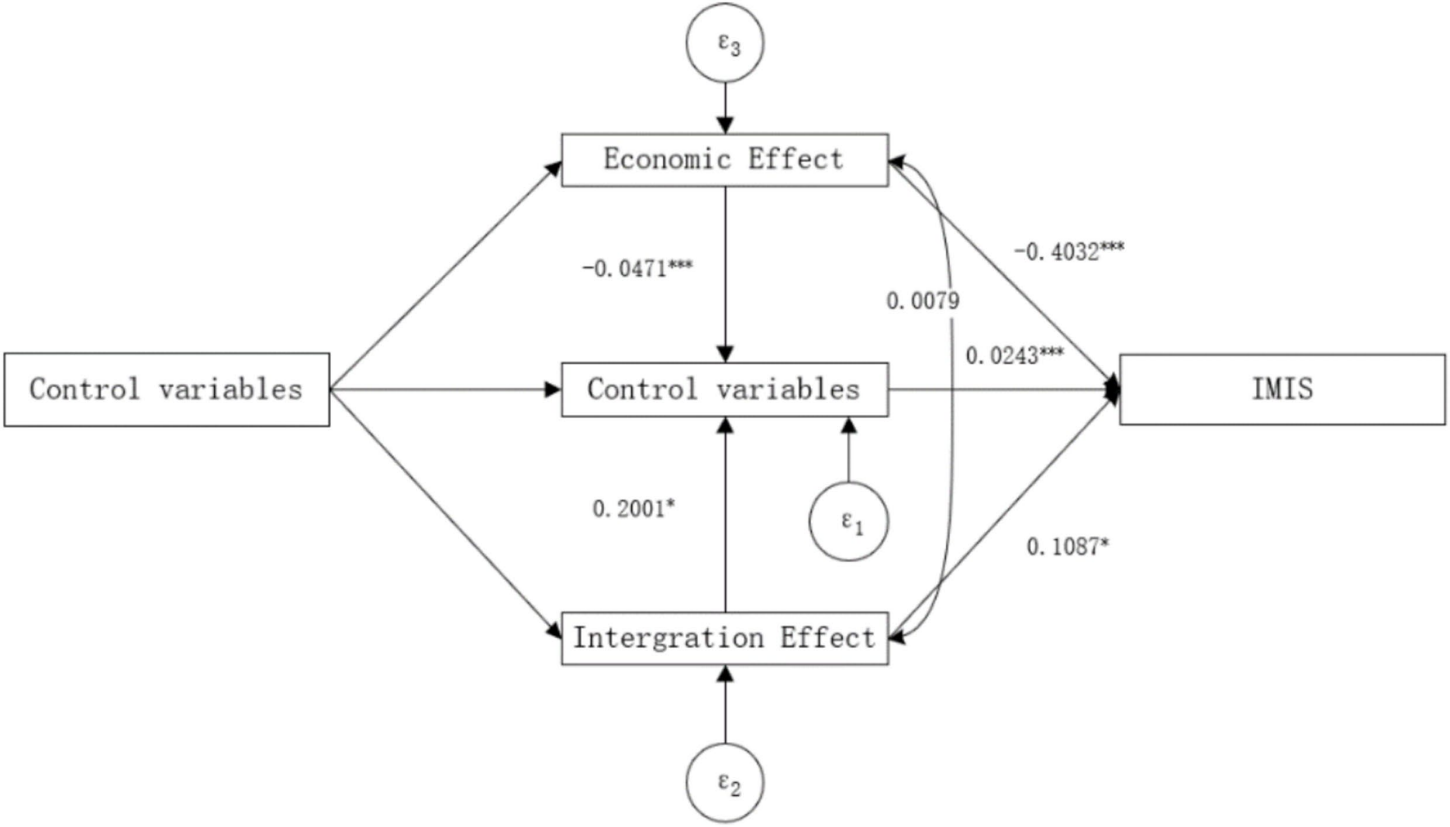
Mediation effects in path models.

Therefore, we employ path analysis within the Structural Equation Modeling (SEM) framework to test these influences. Compared to the mediation model, the SEM helps clarify complex relationships between unobservable mediating variables of different attributes, as well as between mediating variables and control variables, and allows for the assessment of parameter differences across groups with distinct characteristics, thereby eliminating the interference of other factors and enabling reasonable estimation of mediating effects (76, 77). As shown in Figure 4, even after incorporating pathways for economic and integration effects, the coefficient for the IMIS variable remains positive and significant at the 1% statistical level, indicating that IMIS can directly impact the happiness of returning migrant workers, with both effects playing a partial mediating role.

#### 5.1.1 Economic effects

The economic effects posit that the IMIS can influence the happiness of returning migrant workers by reducing their medical burden. Therefore, we examine the changes in individual medical burdens before and after the implementation of IMIS. Medical burden is primarily measured by the out-of-pocket (OOP) hospitalization expenses^3^ incurred by returning migrant workers within a year. The results shown in Figure 4 indicate that IMIS has a significant negative impact on the OOP hospitalization expenses of returning migrant workers. Additionally, there is a significant negative impact of these expenses on their happiness. Combined with the direct effect of IMIS on the happiness of returning migrant workers, the economic effects act as a partial mediator in this relationship. The mediation effect accounts for 25.59% of the total effect (−0.4032 × −0.0471 / 0.0742). These findings suggest that the IMIS policy has increased subsidies for rural healthcare and improved inpatient coverage, thereby reducing the hospitalization burden compared to the NRCMS period. This alleviation of the “high cost of medical care” issue has increased disposable income, thereby enhancing the happiness of returning migrant workers.

#### 5.1.2 Integration effects

Returning migrant workers often experience reduced happiness due to the perceived urban–rural disparity from their work experiences outside the rural areas. This perception of disparity frequently results in their decision to settle in urban areas (62). The integration effects posit that the IMIS can enhance the sense of fairness among returning migrant workers, thereby increasing their willingness to reside in rural areas. Previous research has shown that migrant workers have strong ties to their hometowns, and living in their native rural areas can alleviate stress compared to moving to cities, thus enhancing their happiness (60). This section examines changes in the willingness of returning migrant workers to reside in rural areas before and after the implementation of IMIS.

The willingness to reside in rural areas is primarily measured by the question, “Do you plan to settle in urban areas in the next 5 years?” A response of “No” indicates that returning migrant workers still want to live in rural areas and is coded as 1, while a response of “Yes” is coded as 0. The results in Figure 4 show that IMIS has a significant positive impact on the willingness of returning migrant workers to reside in rural areas. Additionally, the willingness to reside in rural areas has a significant positive impact on their happiness. Considering the direct effect of IMIS on the happiness of returning migrant workers, the integration effects act as a partial mediator in this relationship, with the mediation effect accounting for 29.31% of the total effect (0.1087 × 0.2001 / 0.0742).

These findings indicate that IMIS has reduced the urban–rural disparity and improved the perception of fairness among returning migrant workers. As a result, they are less likely to move away from rural areas due to the significant urban–rural gap, and their strong ties to their hometowns enhance their happiness.

### 5.2 Heterogeneity analysis

#### 5.2.1 Age cohorts

In recent years, an intergenerational shift has been observed within the migrant worker population. Due to differences in upbringing, educational background, and ideological perspectives, older and newer generations of migrant workers may react differently to the same issue (29). Based on this, we divided the sample into those born before 1980 and those born in 1980 or later. The empirical results are presented in Table 5 Panel A. We find that the impact of IMIS on the happiness of returning migrant workers born before 1980 is not significant. In contrast, for those born in 1980 or later, the impact is positive and significant at the 1% statistical level, indicating that IMIS mainly enhances the happiness of the new generation of returning migrant workers. Two reasons may explain this finding: Firstly, compared to the older generation, the new generation of migrant workers has more exposure to the internet and emerging social media, which allows them to stay updated on various policy changes and increases their understanding and perception of these policies.

**Table 5:** Heterogeneity results.

Variables	(1)	(2)
Panel A. Subgroups by born year		
	Before 1980	After 1980
IMIS	0.1651	0.6169***
	(1.60)	(4.86)
Observations	3,370	1,246
Panel B. Subgroups by income level		
	High	Low
IMIS	0.3309***	0.1484
	(3.23)	(0.77)
Observations	2,136	2,480
Panel C. Subgroups by health human capital		
	High	Low
IMIS	0.5460***	0.0472
	(4.61)	(0.33)
Observations	2,396	2,220
Panel D. Subgroups by reimbursement levels		
	High	Low
IMIS	0.1454	0.5022***
	(1.15)	(2.38)
Observations	2,854	1762
Control Variables	Yes	Yes
Year FE	Yes	Yes
City FE	Yes	Yes
Year×City FE	Yes	Yes

***, **, and *, respectively, represent significant estimation coefficients at the 1, 5, and 10% test levels; The value of t is enclosed in parentheses.

Secondly, the new generation of migrant workers is in a critical phase of their life journey, facing higher risks in both work and life. Improved medical insurance can significantly reduce potential future losses due to these risks. In contrast, the older generation tends to have more stable lives and more cautious labor supply choices, resulting in a lower probability of encountering risks and thus weaker perception of changes in medical insurance benefits. Therefore, the happiness effect of IMIS is stronger for the new generation of returning migrant workers.

#### 5.2.2 Income levels

We divide the returning migrant workers into high-income and low-income groups based on their average annual income to examine the effect of IMIS. The results are presented in Table 5. The findings reveal that IMIS has no significant impact on the happiness of the low-income group. However, for the high-income group, the impact is positive and significant at the 1% statistical level, indicating that IMIS primarily enhances the happiness of high-income returning migrant workers. This result may be attributed to a “pro-rich” reverse effect produced by IMIS. Due to the unequal distribution of medical resources and restrictions imposed by reimbursement ratios, deductibles, and caps, wealthier individuals, with their stronger financial capacity, can access more and higher-quality medical resources, resulting in a “the poor subsidizing the rich” phenomenon in medical services (78). In other words, the improvements brought by IMIS are more likely to benefit the high-income group, thereby enhancing their subjective happiness. This phenomenon may be more pronounced in regions that have implemented a multi-tiered system.

#### 5.2.3 Health human capital

The level of human capital is a crucial factor affecting individual happiness (79). Additionally, health status is a vital physical carrier of happiness, and self-rated health can reflect an individual’s subjective feelings, closely related to happiness (80). This study categorizes returning migrant workers based on their self-rated health status, with “very unhealthy” and “somewhat unhealthy” classified as the low health human capital group, and all others classified as the high health human capital group. The empirical results are shown in Table 5 Panel C. The findings indicate that IMIS has no significant effect on the happiness of the low health human capital group. In contrast, for the high health human capital group, the impact is positive and significant at the 1% statistical level, suggesting that IMIS mainly enhances the happiness of returning migrant workers with high health human capital. The underlying reason might be that health human capital is often positively correlated with income. Low-income groups face opportunity loss and capability deprivation due to poor health, leading them into a “poverty trap,” which continuously diminishes their ability to earn income and access medical resources (81). Consequently, they cannot promptly perceive the subjective happiness effects brought by IMIS. On the other hand, individuals with high health human capital generally have higher incomes and can access more medical resources, making them more sensitive to the welfare effects of IMIS, thereby significantly enhancing their happiness with improved healthcare benefits.

#### 5.2.4 Regional average reimbursement levels

This study divides the sample regions into high and low reimbursement level areas based on the average reimbursement level of the regions where the returning migrant workers reside, examining the effect of IMIS on their happiness. The empirical results are presented in Table 5 Panel D. The findings indicate that IMIS has no significant effect on the happiness of groups in high reimbursement level areas. However, for those in low reimbursement level areas, the impact is positive and significant at the 1% statistical level. This suggests that IMIS primarily enhances the happiness of returning migrant workers in low reimbursement level areas. The possible reason for this is that in high reimbursement level areas, the medical needs of returning migrant workers may have already been met to some extent prior to IMIS. Additionally, these areas might experience relatively smaller urban–rural disparities, making returning migrant workers less sensitive to the improvements brought by IMIS. In contrast, in low reimbursement level areas where medical needs are yet to be adequately met, IMIS can significantly alleviate their medical burden and enhance their perception of fairness, thereby improving their happiness.

## 6 Discussion

This study uses the four-wave panel data from the CLDS spanning 2012 to 2018. By employing the gradual reform of the IMIS as a policy shock, we systematically examine the impact of this integration on the happiness of returning migrant workers through a DID model. Robustness checks are performed through various methods to ensure the reliability of our findings. Furthermore, using structural equation modeling, we investigate the mediating mechanisms and analyze the heterogeneous characteristics affecting the impact.

The findings of this study demonstrate that the integration of IMIS has a significant positive impact on the happiness of returning migrant workers. This result remains robust even after a series of sensitivity and robustness checks. To the best of our knowledge, no prior research has specifically examined the relationship between IMIS and the happiness of returning migrant workers in China. However, several studies conducted in different contexts have shown that health insurance systems can play a crucial role in improving individuals’ subjective happiness (11, 82, 83), indirectly supporting our findings.

Furthermore, when distinguishing between different subgroups, we found that the impact of IMIS varies across generational cohorts. Specifically, the results indicate that the improvement in happiness is more pronounced among younger returning migrant workers, whereas the effect is not significant for the older generation born before 1980. This finding aligns with Cheng and Hua (29), who observed that the impact of social insurance on happiness is greater for the younger generation of migrant workers compared to their older counterparts. The generational heterogeneity may stem from differences in the risks faced by these subgroups, as well as their exposure to and understanding of policy changes. On the one hand, younger migrant workers are in the critical stages of their careers and face higher occupational and life risks, making them more sensitive to changes in health insurance benefits. In contrast, older migrant workers tend to have more stable lives and make more cautious employment choices, thus facing lower risks and responding less to changes in insurance coverage.

Therefore, for the younger generation, IMIS plays a more effective role in mitigating their future economic and health risks (84). On the other hand, younger migrant workers tend to use social media more frequently, which allows them to access and understand changes in national policies more promptly. This increased awareness and utilization of policies enhances their sensitivity and efficiency in benefiting from IMIS (85). The deeper understanding and more effective use of these policies may further amplify the positive impact of IMIS on their happiness. Compared to the new generation of migrant workers, the older generation of migrant workers have less access to social media, resulting in a relative lag in policy perception and utilization, and a lack of the necessary sensitivity and efficiency. This limits their ability to fully utilize the opportunities and benefits offered by IMIS policies, which in turn affects the significant increase in their happiness.

The heterogeneity in income levels reveals a “pro-rich” bias in the effect of IMIS on improving happiness. Specifically, the implementation of IMIS has a significantly stronger effect on the happiness of high-income returning migrant workers compared to their low-income counterparts. Similar findings have been observed in other rural populations, with Liu et al. (86) reporting that IMIS significantly enhances the happiness of high-income older adult in rural areas. This phenomenon is not surprising. First, due to the unequal distribution of medical resources, as well as the existence of reimbursement rates, deductibles, and coverage limits, high-income individuals are better positioned to access high-quality healthcare resources through their stronger financial capacity. IMIS, by providing comprehensive coverage and higher reimbursement rates, reduces the additional financial burden associated with obtaining high-quality medical care for wealthier individuals (69), thereby significantly enhancing their happiness.

In contrast, while low-income individuals experience some relief in financial burden after the integration, their limited ability to manage high medical expenses results in a relatively smaller improvement in their happiness (57). Moreover, high-income individuals tend to have higher expectations for the quality and convenience of medical services. The improvements in service quality and accessibility brought about by IMIS may thus have a more pronounced impact on the happiness of wealthier groups. On the other hand, low-income groups are primarily concerned with basic healthcare coverage and tend to have lower expectations regarding service quality. Therefore, the improvements in medical service quality under IMIS may have a more limited effect on their overall happiness.

The heterogeneity in health human capital highlights the varying effects of IMIS on subjective happiness across different health capital groups. Specifically, we find that the implementation of IMIS has a more pronounced positive impact on individuals with higher health human capital. This can be attributed to their stronger competitiveness in the labor market, which enables them to access more job opportunities and earn higher incomes (87), ultimately accumulating more social capital and economic resources. These advantages allow them not only to be more responsive to changes in health insurance policies but also to make more effective use of healthcare benefits, reducing the financial burden of medical expenses. In contrast, individuals with lower health human capital, who face prolonged challenges of poor health and limited job opportunities, are significantly constrained in their ability to work and generate income (81).

The intertwined effects of poor health and poverty weaken their capacity to build social capital and further limit their access to health insurance information and medical resources. This “health poverty trap” results in their inability to substantially improve their quality of life and happiness, even with the implementation of IMIS. In summary, this heterogeneity underscores the shortcomings of IMIS in promoting health equity. It serves as a reminder to policymakers that the challenges and specific needs of individuals with lower health human capital must be addressed when refining policies, to ensure equitable improvements in happiness across different health capital groups.

The heterogeneity in regional reimbursement levels may reflect the relative effectiveness of the policy in relation to existing healthcare coverage across regions. Specifically, we find that the effect of the IMIS on enhancing the happiness of returning rural migrant workers is more pronounced in areas with lower reimbursement levels. This difference may be attributed to the diminishing marginal returns of IMIS. In regions with low reimbursement levels, residents’ healthcare needs are not fully met, offering greater room for welfare improvement through the policy. By raising reimbursement rates, IMIS significantly alleviates the economic burden on residents and addresses long-standing unmet healthcare needs, resulting in a substantial boost to happiness. In contrast, in regions with higher levels of healthcare coverage, where residents’ healthcare needs are relatively better met, the marginal benefits of IMIS diminish, and its impact on happiness becomes less significant. This heterogeneity underscores IMIS’s potential to reduce regional disparities in healthcare resources and promote balanced development. However, it also highlights the need for policymakers to recognize that in areas with high reimbursement levels, the marginal effects of the policy are lower, necessitating considerations on how to optimize resource allocation to ensure the policy generates greater benefits across all regions.

We further explore the mechanisms by which the IMIS enhances the happiness of returning migrant workers. The results suggest that the economic effect and integration effect are the potential driver of this result. Specifically, the economic effect refers to the significant reduction in hospitalization costs for returning migrant workers due to IMIS, which in turn boosts their happiness. According to consumer behavior theory, under certain budget constraints, consumers allocate and choose between healthcare consumption and other goods. IMIS effectively reduces the relative price of healthcare services (88), altering the original budget constraint and increasing consumers’ consumption of other goods, thereby enhancing their happiness. This causal chain also explains the heterogeneity based on income levels.

For low-income groups, although the relative price of healthcare services decreases, their lower income levels limit the extent to which they can increase consumption of other goods, thus constraining the improvement in their happiness. This phenomenon does not apply to high-income groups, where IMIS has a greater positive effect on happiness. In fact, Liu et al. (86) found that IMIS significantly reduced residents’ medical expenses. Additionally, Finkelstein and McKnight (89) noted that a reduction in medical expenses can significantly improve individual happiness. Our study not only corroborates these findings but also strengthens the causal relationship between economic effects and happiness improvement through empirical analysis, thereby reinforcing the complete causal chain.

The integration effect indicates that returning migrant workers’ willingness to reside in rural areas plays a crucial mediating role in the impact of the policy. This willingness to stay in rural areas may reflect perceptions of urban–rural equity. When the urban–rural gap is significant, the superior living conditions in cities often attract migrant workers to reside in urban areas long-term, rather than returning to rural regions. The IMIS narrows the gap in healthcare services between urban and rural areas, reducing the appeal of urban benefits for migrant workers, thus increasing their willingness to reside in rural areas (69, 74). Wang et al. (74) further noted that this phenomenon is more pronounced among the younger generation of rural residents. This may be due to the fact that younger individuals are at a critical stage of life and work, facing greater instability, and are more exposed to new experiences, making them more responsive to changes.

Moreover, Li and Wang (90) argued that when objective living arrangements align with subjective preferences, happiness is enhanced. Therefore, IMIS can enhance the happiness of migrant workers, particularly the younger generation, through this integration effect. While we provide this explanation, it is important to acknowledge that perceived fairness is difficult to quantify directly. Thus, this mechanism does not imply a strict causal inference, but it offers important insights for policymakers. The IMIS has the potential to reduce the urban–rural gap, enhance migrant workers’ sense of fairness, and increase their willingness to stay in rural areas, ultimately improving their overall happiness.

## 7 Conclusion

Based on the results of this study, we offer several policy recommendations to guide future reforms. First, in advancing the integration of the IMIS, it is essential to prioritize flexibility in the system to avoid a one-size-fits-all approach. Policy adjustments should be made incrementally, tailored to local economic conditions and available healthcare resources. By refining policy design, the efficiency of health insurance fund utilization can be improved, particularly through higher reimbursement rates aimed at alleviating the financial burden of medical expenses. Second, increasing investment in rural primary healthcare is essential. By improving medical facilities and enhancing the healthcare workforce in rural areas, the urban–rural disparity in healthcare resources can be reduced, thereby improving both satisfaction with healthcare services and the perceived fairness among returning migrant workers. Third, targeted identification mechanisms should be established to focus on vulnerable groups, such as older adults, those with poor health, and low-income rural residents. Drawing on the U.S. Medicaid program, more lenient payment policies and additional financial subsidies should be provided to these groups. Finally, tailored policy promotion campaigns are necessary to ensure that different age groups receive accurate and comprehensive information about healthcare policies. By utilizing both new and traditional media, the dissemination of healthcare policies can be more effective, ensuring that the benefits of integration reach all segments of the population.

This study has several limitations. Firstly, although efforts were made to address endogeneity issues, there are inherent drawbacks associated with the IVs used. On the one hand, IVs are not unique, which introduces a degree of arbitrariness in their estimation. On the other hand, since the error term is inherently unobservable, identifying variables that are strictly uncorrelated with the error term but highly correlated with the endogenous explanatory variables is challenging. In other words, due to data constraints, the selected IVs may not be entirely independent of the error term, potentially leading to an overestimation of policy effects. Although we employed the System-GMM method for further validation, this approach may still fall short of fully resolving the issue. Therefore, exploring more effective methods to address endogeneity in IMIS remains a key focus of our future research.

Secondly, the internal mechanisms through which IMIS affects happiness are likely complex and multifaceted due to the influence of various factors on perceptions of happiness. The economic and integration effects proposed in this study represent only part of these mechanisms and do not capture all possible pathways. For instance, existing studies indicate that health (both subjective and objective) significantly impacts individual happiness, with healthier individuals reporting higher levels of happiness (91). As a health insurance reform policy, IMIS directly affects individuals’ health, suggesting that health could also serve as an intrinsic mechanism through which IMIS influences happiness. However, due to data limitations, empirical testing of this pathway is currently challenging. A more comprehensive exploration of the mechanisms underlying the impact of IMIS on happiness has thus become a primary direction for future research.

## Data Availability

The datasets presented in this study can be found in online repositories. The names of the repository/repositories and accession number(s) can be found at: China Labor-force Dynamics Survey (CLDS) https://isg.sysu.edu.cn/node/353.

## Footnotes

^1^It is the National Development and Reform Commission’s estimate based on the sample survey, available from http://www.xinhuanet.com/finance/2018-07/25/c_1123176883.htm

^2^Given the potential impact of endogeneity on the validity of the results and the inertia inherent in changes in happiness, where prior happiness levels may influence current levels, we introduce lagged happiness (one period) and employ the System-GMM method for parameter estimation. This approach is commonly used to address endogeneity issues and ensures the robustness of the analysis. The results show that the coefficient for IMIS is 0.1358, statistically significant at the 5% level. These findings suggest that, after adequately addressing endogeneity, IMIS significantly enhances the happiness of returning migrant workers.

^3^The out-of-pocket (OOP) hospitalization expenses are logarithmically transformed to mitigate estimation bias due to data fluctuations.
